# Book Reviews

**Published:** 1808-10

**Authors:** 


### CRITICAL ANALYSIS
### OF THE
### RECENT PUBLICATIONS
### ON THE
### DIFFERENT BRANCHES OF PHYSIC, SURGERY, AND MEDICAL PHILOSOPHY.

---

*A General View of the Natural History of the Atmosphere, and of its Connection with the Sciences of Medicine and Agriculture; including an Essay on the Causes of Epidemical Diseases.* By HENRY ROBERTSON, M. D. Edinburgh, 2 vol. 8vo. 1808.

THE importance of the subject, announced in these Volumes, is every day more strongly confirmed; and it would be ungrateful not to acknowledge our obligation for the diligence evinced by the author. We shall generally announce the contents of the whole, and analyze only that part of it which is more immediately connected with the inquiries of our readers.

The first volume opens the subject with an account of the general properties of light, heat, and electricity. After this follows a chapter on the physical properties of the atmosphere, under the description of its colour, fluidity, density, and elasticity, including some remarks on akoustics.

The gravity of the air, variations of the barometer, and their causes as far as ascertained. Temperature of the atmosphere and causes of varieties of climate, &c. Supposed change of climates in certain countries.

Meteorology is next considered, compredending spontaneous evaporation and its causes. Rain, snow, hail, water-spouts, and other meteors connected with the existence of water in the atmosphere—of winds—of luminous meteors, of the rainbow, of the parhelion, au parselene, or mock-sun and moon, of halos,—of electrical meteors, fire balls, shooting or falling-stars, aurora borealis,—of phosphorescent meteors, ignis fatuus,—thunder stones, —prognostics of the weather.

The second volume commences with a chapter on the chemical properties of the atmosphere, and comprehends, besides general remarks, eudiometry—combustion—substances formed by the combination of oxygen and azotic gases—oxygen gas—nitrogen gas—carbonic acid gas—combination of the constituent gases in the formation of the atmosphere—respiration—the temperature of living bodies. The influence of the atmosphere on growing plants, germination, vegetation,—the influence of the atmosphere on animal and vegetable remains.

These considerations lead our author to some general observations on the influence of climate on animals and plants, as well as the influence of atmospheric temperature, and different states of gravity, vity, of the variations of humidity, and different degrees of electricity.

The last chapter is on the influence of extraneous matters in the atmosphere on animals and plants. As we presume most of our readers have paid sufficient attention to the more established laws of the atmosphere, we shall confine ourselves to the author's remarks on epidemic contagion and marsh effluvia.

In considering the causes of contagion, the author's first object is to investigate the supposed influence of the atmosphere in *engendering* epidemical diseases; next, to enquire into those matters which are supposed to have the same powers; and, thirdly, to consider the influence of the atmosphere in propagating their effects.

It is first remarked, that no author who has imputed epidemical diseases to a peculiarity in the nature of the atmosphere, has ever attempted to describe the supposed alteration in the qualities of the air to which the disease may be attributed. "Parey, we are told, imputes the occurrence of plague to an alteration in the natural properties of the air, without giving a precise definition of them; but as he also talks of its putrefaction as arising from the exhalations of other matters, his opinion cannot be therefore regarded on this subject as decided. But that a change of the properties of the air, independent of the mixture of any accidental impurities, was generally esteemed as giving origin to epidemical disorders, and continues down to the present time, may be collected from many authors who have written on this subject. The following quotation from Mindererus is therefore selected, as affording the most distinct view of this opinion: he says, when treating of the plague, 'Longe porro communior pestis causa esse solet aër; non is veneno quidem aut putredine vitiatus, sed inordinata primarum qualitatum subsecutione noxius, utpote cum is modo in calidum, modo in frigidum, aut siccum declinans, fortuito quasi eventu, multoque temporis habito respectu ac ratione, præpostero plane ordine, nunc in hieme calet, nunc friget in æstate.' Dr. Maclean, in the Appendix to his View of the Science of Life, p. 20, in treating of this question, observes, 'Certain states or vicissitudes of the atmosphere, together with the application of other powers, producing indirect debility, are the cause of all epidemic and pestilential diseases which affect the same person more than once during life, and have hitherto been reputed contagious. In page 180, of Dr. Arbuthnot's Essay on the Effects of Air, this learned physician advances the following opinion respecting this question: 'Whether the air is capable of producing the plague without infection, in a place where it is not an endemical disease? I think the affirmative is very probable; for, 1st, In a country where it is both an indigenous and endemical disease, it must be probably owing to some particular quality of the air; 2dly, It has been proved, that there is hardly any alteration, even to the utmost degree of putrefaction, but may be produced by the enormities, combinations, and alterations of the common qualities of the air; and that still more extraordinary traordinary effects may be produced from some contagion from the air, by uncommon effluvia from bodies near the surface of the earth.' The greater part of the chapter contains arguments in support of this doctrine; but, like almost every author who has assumed this side of the question, the Doctor is by no means sufficiently accurate in making a distinction between the natural effects of the changes of the physical properties of the atmosphere, and of the effects of such matters as may be occasionally mixed with it. In Dr. Adams's elaborate work on Animal Poisons, the same obscurity pervades the investigation of this subject: in page 320, he mentions, as a cause of fever, 'a state of the air in its cause, unconnected with living animals, but equally affecting all who come within its influence, as intermittents from marsh miasmata, influenza, or any other endemic or epidemic disease.' In page 354, of the same work, he continues, 'If what we have said be admitted, it will follow, that fever from infectious atmosphere is to be ascribed to the confined effluvia from living bodies,—the yellow fever from the effluvia of putrid animal or vegetable matter exposed to a hot sun, and the plague to the latter, joined to an epidemic constitution of the air; that in all, a *fomes* may excite the disease; yet we are not certain, whether a diseased subject is contagious, unless in a certain atmosphere.' In such terms, he admits the existence of contagion, and seemingly attributes a power of equal influence to the atmosphere also.

"But the effect that may be supposed to be produced by any alteration of the atmosphere in the manner thus pointed out, would certainly rather tend to excite such diseases as generally arise from variations of the climate. It is impossible to comprehend, how a change in the physical properties of the atmosphere, capable of engendering any general disease, should take place, without other circumstances connected with that change upon other bodies becoming manifest. Were the above opinion correct, this must have been always the case, considering the variety of such diseases. Neither is it easy to conceive, how an alteration in the constitution of the air, rendering it so virulent as to occasion any pestilential disorder, should not affect every one at nearly the same time; as, in every condition of the atmosphere, it must act upon man, and all respiring animals, in nearly a similar manner: therefore, did any epidemical malady really originate from this cause, few or none could escape its malignity; and the brute creation must have been in that case equally afflicted by it as mankind. But Plenciz assures us, from the result of long experience, that the epidemical diseases of cattle are rarely or never communicated to their herds, or even to their dogs: in this general observation, hydrophobia is to be excepted. On the other hand, it requires no authority to demonstrate, that the diseases of man are never transferred even to domestic animals. A full investigation, in proof of these remarks, is to be found in the preface to Muratori's work on the Management of the Plague."

It

It is impossible not to arrest our readers, in order to set Dr. Robertson somewhat to rights in this passage. If Parey imputes the occurrence of plague to an alteration in the properties of the air, he only does so because he could find no other cause; nor was it by any means to be expected that he should ascertain what that change is, any more than what is the nature of small-pox, or other contagious effluvia. Mindererus seems to acknowledge himself equally in the dark, or else to cover his ignorance by the obscurity of his terms. Maclean, perhaps, speaks with too much authority, but without pretending to detect causes for what is not yet the subject of experiment. The accusation against Dr. Arbuthnot seems more reasonable, as it must be admitted that he has confused what may be a mere production of the air with what may be floating within it. But in the quotation from Dr. Adams, we find a distinction made between a bad state of the air, *unconnected with living* animals, air rendered infectious *from the effluvia of living* animals, and air *impregnated with the effluvia of dead animal or vegetable matter exposed to a hot sun*, and the combination of the latter with a *pestilential state of the atmosphere*. It is remarkable too, when this author doubts whether the diseased subject is contagious, but admits an infectious property in the air, and, consequently, in substances imbued with it, that Dr. Robertson should draw so strange a conclusion, and in terms so little intelligible. "In such terms, says our author, he admits the existence of contagion, and seemingly attributes a power of equal influence to the atmosphere also."

The last conclusion, that the diseases of man are never transferred to brutes, and vice versa, however strengthened by Muratori, comes in most unfortunately for Dr. Robertson, in whatever shape he may please to put it. If he wishes to prove that the atmosphere cannot be deleterious without affecting brutes also, it cannot be necessary to remind him, that many pestilential diseases have begun with cattle; and as to contagions, we need not remind him of the cow-pox, as he himself admits hydrophobia.

Some further remarks follow, on the dreadful epidemic which desolated Gibraltar and other parts of Spain. This the author imputes, but we think without sufficient authority, to a communication of the infected with other districts. How much it is to be regretted, that our accounts of this most important event, are still so imperfect. As to what is said of the partial manner in which individual cantons escaped the disease, we cannot help thinking this would tend to prove that the whole must depend on the peculiarities of the air, because it is inconceivable that such districts should be more religiously preserved from intercourse with the infected, than others which have suffered.

The author next adverts to seasons, and shows that the plague has appeared in all seasons, in different parts of the world. This should have been stated with more discrimination. In those parts of the world in which the seasons are regular, the plague has usually ally begun in spring; and in Egypt, always ceases at Midsummer. In England it has usually begun in autumn, and its cessation has been as uncertain as might be expected in such a climate as ours. If the commencement of plagues has been sometimes dated in the winter, there is reason to believe they were of a different character from the true plague, and only acquired their name from their epidemic and destructive properties.

Some remarks follow, on the opinions of various writers, who have denied the contagious property of the plague. Noah Webster, it is said, concludes his remarks in terms similar to those who have thought differently.—His opinion, therefore, goes for nothing with our author; and as Assalini gives a precise account of the great pains he took to preserve himself, this, we are told, shows the absurdity of his opinion, that such pains were unnecessary. Further remarks follow, on Dedier, Chiconeau, and Moseley. In answer to all these, illustrations are brought of the manner in which particular classes or orders of society were obnoxious to, or escaped other diseases which are admitted to be contagious. We could have been glad if our author had produced any race, order, society, or age, or more than a few solitary instances of idiosyncracy, to whom small-pox had not been as general, and even destructive as to any other part of mankind. A quotation follows from Dr. Russel, whose opinions on this subject are well known, and must ever be respected.

Several instances are selected from a variety of authors, historical and medical, to show, that from vapours and effluvia fevers have arisen; but we are not informed on the authority of the same authors, whether the subjects, thus infected themselves, became contagious to others, even when the contaminated air has been conveyed by a fomes. Several instances, however, follow, of "disorders resembling the very worst forms of epidemics which have appeared without the immediate application of one specified cause, but which seem to have been owing to the combined influence of several agents, and to be afterwards propagated by the emanation of a peculiar matter generated in the course of the disorder." In this way, continues our author, fevers of the most malignant description have been generated in besieged towns, ship-board, jails, hospitals, and wherever people are much crowded. We cannot help remarking, that if such a malignant air is spread through every part of a ship, a town, hospital, or jail, it will be extremely difficult to determine whether individuals are infected by each other, or by the general state of the atmosphere. This part of the subject concludes with some remarks, that the original type of a fever, excited by miasma, is sometimes altered, so as to become infectious. If we understand our author, his meaning is, that any epidemical or endemical disease, by the accumulation of numbers of sick, will produce such a change in the air, as to render it injurious, and excite the hospital or jail fever in those who breathe it. All this is undeniable, but it does not necessarily follow, that such such sick individuals, freed from their clothing and well washed, or rather that individuals who have sickened by remaining so short a time in this air, that their clothes would not become sufficiently imbued to prove a fomes, should, by their own secretions, become infectious. And it must be admitted, that no combination of animal effluvia, under any other circumstances of disease, has been accused of generating small-pox, or any other known and universally admitted contagion.

Having thus examined what has been done by his predecessors, Dr. Robertson proceeds to give his own opinions of the sources of epidemic diseases. The first of these, which he considers as most immediately inimical to the living body, is the effluvia arising from the remains of putrid animal matter in a certain state of decay. Of the danger of this, however, he gives no particular instances.

"The second source, continues Dr. Robertson, that gives rise to matters which produce general epidemic diseases, is an accumulation of the usual exhalations from the living body. These being confined within a limited space, enter into new combinations, generating a matter of the most noxious properties. This appears likewise in an aëriform state; and there are frequent instances of it having almost instantly deprived people of life. This appears from the relation we have of the Black Assize at Oxford, and the dreadful sufferings of those confined in the black hole at Calcutta. It is probably owing to the co-operation of this and the former causes, that epidemics of the worst species are generated; and, except in a few instances, this, perhaps, never acts alone: and when combined with other causes, the plague is even generated in places otherwise favourable for its appearance. The contagious diseases that break out in besieged towns likewise appear in consequence of their union. At the same time, there is every reason to suppose, that this cause also frequently acts as a solitary agent. In this way, it is to be considered as the cause of an endemic or local disease."

On this passage, we shall only remark, that; of the Oxford Black Assize, the most authentic accounts assure us that no persons were taken ill excepting those who were in court; that is, that those who were infected by the pestilential effluvia, did not infect any of those who nursed or attended them, nor indeed any other person. What the former causes, mentioned by our author, allude to, we pretend not to determine.

The third, and most pregnant source of epidemic disorders, we are told, is the matter arising from persons under particular diseases. "It is to [by] contagion alone, that certain specific diseases are produced, as small pox, scarlet fever, typhus, plague, &c. Here again we feel ourselves much at a loss, because, though we have every certainty that can be required, that the small-pox cannot be produced but by its own contagion; and that its own contagion will almost invariably produce it in those who have not previously passed through it: yet of the plague we have no such certainty. Of the plague also, we are satisfactorily informed that the the having once passed through it, is no security against a future attack; and also that a diseased subject is never contagious, but at certain seasons, and in certain places.

We have made thus free with this part of Dr. Robertson's work, because the subject is so highly important as to deserve particular attention to every thing connected with it. While, however, we thus differ in opinion from our author, we are ready to give him great credit for the assistance we have received from him, in his numerous references, and the diligence which he has evinced in every other part of his undertaking.

---

### *Dr. Willan, on the Skin.*

( Concluded from our last. )

ORDER IV. "Bullæ (Bleb,) definition XI, (so we have copied from the passage before us, but among the definitions it will be found IX) a large portion of the cuticle detached from the skin by the interposition of a transparent watery fluid. Soon after the water is discharged, the excoriated surface is covered with a flat yellow, or black scab, which remains till a new cuticle is formed underneath. When *Bullæ* have a dark red livid base, they are denominated *phlyctenæ.*"

Of this order, continues Dr. W. I have constituted three genera, Erysipelas, Pemphygus, and Pomphylox. Whether the two latter might be properly united; or whether the Pemphygus should be considered a distinct idiopathic eruptive fever, I do not wish absolutely to decide, but wait for the opinion of judicious critics.

Every one must be pleased with the modesty of this last paragraph.—But we believe most readers, like ourselves, have been in expectation that the question would be solved by the systematic writer whose accuracy we have so often admired, and whom, in the progress of his labours, we have followed till we see him arrived at *Pemphygus.* We shall not, however, anticipate what will naturally suggest itself in the course of our remarks on this number. In these we shall be less reserved, because our known regard for the talents of Dr. Willan, and the useful manner in which he has employed them, will free us from any suspicion of severity; and, because, as we before remarked, the nature of our publication will afford him an opportunity of setting us right if we mistake him.

"The erysipelas is defined a febrile disease, in which some part of the body is affected externally with heat, redness, swelling, and vesications. The tumour is soft, diffuse, and irregularly circumscribed; the redness disappears on pressure, and immediately returns when the pressure is removed."

"Medical and chirurgical writers generally agree in their statements of the characteristics of erysipelas. Ancient authors, however, appear more anxious to distinguish it from phlegmon, than to give a minute history or description of it. According to Galen," heat and tumefaction are symptoms common to both diseases, but

 the the colour of them is different: that of phlegmon being a vivid permanent red; that of erysipelas, a pale red, inclining to yellow, easily obliterated by the touch of the finger. In phlegmon, the tumour is *circumscribed*, hard, tensive, and painful: in erysipelas, it *extends diffusely* with considerable heat and uneasiness, but without tension or throbbing. Phlegmon is seated deep in the flesh, whereas erysipelas does not penetrate far below the skin.—He adds, that erysipelas is in some cases attended with ulceration; that in others it has a tendency to gangrene, and he thinks with Hippocrates, that it often affects internal membranes as well as the skin." To this account of Galen, is added a note containing his distinctions, founded on his theory of the four humours; but the most important part, is his remarks of the manner in which Erysipelas sometimes approaches Phlegmon, and Phlegmon Erysipelas. Some observations follow, on Serapion and Avicenna; after which, we are informed, that "without being influenced by the hypothesis or opinion of Galen, it is the author's intention to divide Erysipelas under four varieties; E. phlegmonodes; E. œdematodes; E. gangrenodes; and E. erraticum."

There cannot be a doubt that an author of Dr. Willan's judgment and celebrity, has an undoubted right to form his own plan, uninfluenced by Galen or any other writer: but where words are adopted, if they have already an appropriate meaning, that meaning should be referred to; or if they are intended as arbitrary terms, the reader should be apprised of the author's object. Galen's distinction between phlegmon and erysipelas was quoted, we presume, for some purpose, probably to guide the reader in comprehending the term phlegmonodes. If so, he should have been informed of it.

By this time, we are fearful our readers will suspect us of unnecessary preciseness, and, perhaps, with reason. But it is time we should explain ourselves.

As Dr. Willan's professed object is with the skin, it may be justly urged that phlegmon being unconnected with that membrane, made no necessary part of his work. But it now appears that the laws of inflammation must be somewhat inquired into, in order to ascertain the distinctions alluded to between phegmon and erysipelas, and also the approximations of each. Galen's description is certainly sufficiently accurate, and we should be satisfied with it if we could collect that our author is so. Celsus also, in the passage to which we are referred, has some useful remarks, though less pointed in his description. Both of them agree with most other writers, that the phlegmonous inflammation is deeper than the surface, that the erysipelatous is for the most part superficial. That the former is circumscribed, whilst the latter is diffused, and continues to spread. That the one is disposed to suppuration, which it early shows by a throbbing pain: that the other rarely suppurates; but sometimes, if it remains superficial, ulcerates; or if it has extended deeper, sphacelates.

Here

Here we shall stop for a moment to indulge a few reflections of our own. And, first, let us remark with the celebrated Cullen, how impossible it is to avoid forming some theory, or some generalization of facts, whenever we mean to convey practical knowledge to others. For want of this, without impeaching the accuracy or industry of Dr. Willan in this important division, we fear the student, after perusing the whole, will retain but little, either for want of some established laws, by which he may judge for himself under every various appearance; or, if he cannot trust to his judgment for want of some aphorisms, which may enable him to refer to his memory.

It may be asked, what is all this to the purpose? We answer, that as soon as we attempt to follow our author in his first division, "Erysipelas phlegmonodes," we cannot help wishing to learn some practical *distinction* from himself, concerning *Phlegmon*, to which we suppose this variety of erysipelas makes some approaches; and as we conceive medicine has derived some advantages since Galen's time, we could have wished for *distinctions* somewhat more recent. It is true, that with the ancients, the *terms* phlegmon and erysipelas, were deduced from their comparative heat and colour. Mr. Hunter, who examined every thing as far as his senses would permit, discovered that the difference between these two inflammations, consisted in certain actions, by which one *confined* the disease by forming an adhesion with coagulated lymph, whilst the other, for want of this provision, spreads and throws out only serum. The first, therefore, he called the *adhesive* inflammation. This is the phlegmon of the Greeks, and may be called inflammation, so conducted by the constitution or parts, as to prevent its spreading, or the diffusion of any extravasated fluids. Erysipelas, on the contrary, is confined by no coagulated lymph, and the serum which is extravasated, is diffused into the cellular substance. By erysipelas phlegmonodes then, we conceive must be meant that kind of erysipelas which approaches nearest to the adhesive inflammation, or which soonest assumes that action, and as the phlegmonic, or adhesive, is the most favourable form of inflammation, the business of art should be to conduct erysipelas to that form. This consideration obliges us for the present to pass over a great deal of Dr. Willan, till we meet him again under the subject of treatment. "All the ancient writers says he, except Galen, recommend blood-letting as the principal remedy in erysipelas."

It is enough for us if we have Galen and Celsus to keep company with Mr. Hunter. In the treatment of diseases, the ancients generally had the more violent stages in view. Probably they were rarely consulted in others. In such, blood-letting is often necessary, because, though erysipelas is, for the most part, the consequence of a state which possesses not powers equal to the adhesive inflammation, yet it sometimes arises from a peculiarity of constitution in other respects healthy. This distinction is admirably marked by Celsus. Interdum vel ex *nimia inflammatione* vel

 ob æstus ob æstus unmodicos, vel ob nimia frigora; vel quia *corpus senile* aut *mali habitus* est, cancer * occupat. By this, we see how correctly Celsus had discovered that erysipelas might be merely too high action, which, if continued, must end in gangrene, as he afterwards shows; yet it might also be the effect of an attempt at phlegmonous inflammation in an aged or enfeebled habit. His mode of practice therefore, relates to both.—Oportet *si vires patiuntur* sanguinem mittere.

We have just offered these general remarks on the distinction between the two species of inflammation, to show, that in our opinion the ancients were not much mistaken when they appear more anxious to distinguish erysipelas from phlegmon, than to give a minute history or description of it. For the same reason, Mr. Hunter in his elaborate Treatise on Inflammation, confines himself principally to the adhesive, considering, that by a perfect knowledge of its laws, every deviation from it would be best understood. Erysipelas is the most common deviation, yet the forms of which are so various, that we are obliged to Dr. Willan for tracing them. But under every form, excepting in the single instance alluded to, erysipelas must be considered as increased action without strength to support it. The business of art is therefore to increase the strength of the patient by every means that are not directed to increase action, always keeping in view that as the most favourable kind of erysipelas, is that which approaches nearest to adhesive inflammation; (for such, we conceive, must be the meaning of erysipelas phlegmonodes) so all our endeavours are to be directed to inducing such a change. In order to produce this favourable issue, after blood-letting, *if the patient's strength will bear it*, the practice of Celsus, which was chiefly local, may seem severe, and as it is now out of use, it would be difficult to give an estimate of its success. Mr. Hunter's practice, it appears, was the exhibition of bark internally, in which he was supported by that experienced and sagacious practitioner, G. Fordyce.—Dr. W's extracts from Garthshore, Wells, and others, confirm the same.

Having made these general remarks, which we could not do without in some degree pursuing the disease and remedies through their whole progress, we shall proceed to describe the varieties from our author.

"Erysipelas phlegmonodes, says he, generally affects one side of the face, or one of the limbs. In the former case, the disease begins with coldness and shiverings, which alternate with irregular flushes of heat: on the 2d and 3d days, the pulse is increased in frequency, oppressed, and somewhat hard; the tongue is covered with a whitish crust; there is a sensation of languor, and general soreness, attended with thirst, loss of appetite, and a disposition to

* Cels. Lib. 5, cap. 31. Every reader knows, and the passage referred to teaches us, that erysipelas was with Celsus one species of cancer.

to sleep. Dull aching pains are also felt in the head, neck, and back. The swelling usually appears in the second night, or on the third day, at the side of the nose, and on the cheek, or sometimes near the ear. It is of a dark red colour, smooth and soft, and attended with a sensation of heat and tingling This redness, and the swelling, extend gradually over the side of the face, and spread, in some cases, to the scalp, and to the side of the neck, or upper part of the breast. The face then appears much disfigured; the mouth is drawn to one side; the eye-lids are turgid, and close up the eye; the symptoms of fever become more violent, and are attended with delirium. On the 4th and 5th day, vesications arise on different parts of the diseased surface, especially at the central part of the swelling. They have an irregular base, and are of various sizes. The fluid contained in them is at first clear and watery; it becomes afterwards straw-coloured and opaque, or sometimes slightly livid, without losing its transparency. Soon after the fluid has been discharged from the vesications, the excoriated surface is covered with a yellowish or black irregular scab. The bullæ usually break on the 5th or 6th day, when the swelling begins to subside, and assumes a yellowish hue. At this time also, there is a considerable remission of the fever. On the 8th day, both the fever and swelling generally disappear. I never saw this disease terminate fatally: it is, however, observed by authors, that some patients die in a comatose state on the 7th, 8th, or 9th day. When the redness changes to a brown or yellowish hue, a separation of the cuticle commences at the edges of the tumefaction, and is continued over the whole of it: a new cuticle is formed on the 10th day, and the scabs fall off about the same time."

Some varieties are next marked, and afterwards the disease described in its attacks on the lower extremities: under this last it appears to us, that in attending to other writers, the author has included the Barbadoes leg, or as it is now called, morbus glandularis. Erysipelas œdematodes seems the same as the former, excepting that there is still less disposition to terminate in the adhesive inflammation, in consequence of which, if the disease is violent, it continues to spread, and the serous effusion to increase till the brain is affected. Erysipelas gangrænosum, our readers will perceive, is only a higher degree of erysipelatous inflammation, with less power of supporting it.

Erysipelas erraticum is distinguished by the disease appearing in various parts of the body, under the form of red patches without any certain order. Under this division is placed that species of erysipelas which has been observed by Underwood, Broomfield, Dr. Garthshore, and some others, in young infants, and also those cases in which the inoculated small-pox and cow-pox have proved fatal, from their first insertion as well as some more general appearances after small-pox in the casual way. The question con-

 cerning cerning the contagious property of erysipelas is left undecided by the author, but most of the authorities are produced.

On the subject of remedies Dr. W. very justly observes, that however sizy the blood may appear, it rarely happens but that repeated blood-letting increases the symptoms, and protracts the disease. Many physicians object to blisters; and certainly to cure a disease of vesication, high irritation, and tendency to gangrene, the application of cantharides may appear paradoxical. But Dr. W. very justly observes, that however improper they may be too near the disease, they may often be serviceable in diverting it from the face and scalp Leeches are also advised with the same caution. Saline medicines, preparations of antimony, compound powder of ipecacuanha, are offered for the choice of the reader. Hoffman and others are said to have over valued the effects of camphor and the dulcified acids in preventing too great determination of the blood to the head, and the reader is advised to consult Friend, from whom a long extract is offered on the propriety of repeated purges.

In erysipelas œdematodes, we are directed to employ blisters, diaphoretics, volatile alkali, and purgatives during the first three or four days, after which Peruvian bark and diuretics. Subjoined to volatile alkali is a quotation from Dr. Peart. We have no inclination to doubt the accuracy of that gentleman; but Dr. Willan's readers will expect his own practical remarks; had he informed us of them, it would have been enough to have acknowledged that he was indebted to Dr. Peart for the first hint on this subject.

In erysipelas gangrænosum, and erraticum, the great disposition to mortification points out the necessity of bark, without any question; mineral acids and opium in moderate doses are also recommended, and the general treatment as in malignant fever.

"The Pemphigus, says Dr. W. is an acute disease, characterized by an eruption of phlyctænæ, or vesications with an inflamed base, appearing in succession on different parts of the surface of the body, and sometimes in the mouth. It differs from erysipelas in its progress and duration, but it is more particularly distinguishable from that disease, as it does not exhibit any tumefaction, or redness, of the parts on which the vesications appear.

"The varieties of pemphigus, described by medical writers, may be arranged under the titles, Pemphigus vulgaris, Pemphigus contagiosus, and Pemphigus infantilis. Having never myself seen an instance of either of the former, I cannot do more than state what has been said respecting them by authors."

There is some variety in the description of different authors, as quoted by Dr. Willan; they all, however, speak of solitary cases. Sauvage of one from Seliger, and one in his own practice. Dr. Winterbottom, to us unaccountably, describes his case as occurring in the second day of an intermittent, yet the whole disease ceased as the eruption dried. Dr. Dickson's is contained in in the Memoirs of the Irish Academy. All these cases agree in the figure and size of the vesicle, the progress of the disease, and its favorable issue.

Pemphigus contagiosus is related in a paper by Dr. Langham, translated from the Acta Helvetica, which begins thus:

"Towards the end of the last winter, an epidemical disease of a very peculiar nature, hitherto undescribed and unknown, made its appearance in this country, and prevailed, through the whole succeeding summer, in so violent a degree, that all persons affected by it died. It was so highly infectious, that no person escaped it, who had for any length of time been exposed to the contagion; hence it spread through whole families, with great rapidity."

After this quotation the reader will join with us in praying. "*Di nobis talem avertite pestem*" Happily, however, the Doctor contrived to cure some; for after explaining his mode of treatment he tells us, After health had been restored, I kept the bowels open for some days, &c. Though Dr. Langham thinks his disease has never been paralleled, (and in some respects he is perhaps right) yet Dr. W. very properly remarks, that in malignant fever of all kinds, vesications have been frequently described; and probably, Dr. L's disease was only remarkable for such vesications being more general than usually happens.

"Pemphygus infantilis," as described by Dr. Willan, exhibits irregular oblong vesications or phlyctænæ, of a considerable size, and generally flattened at the top. They are at first small and transparent, but as they enlarge, the fluid contained in them assumes a purplish hue, and finally becomes turbid, from a slight admixture of pus. They are also surrounded by an inflamed border of a livid red colour. This eruption sometimes appears in infants two or three days after birth, on the neck, and upper part of the breast, on the abdomen, the groin, the scrotum, and inner part of the thighs. When the fluid is discharged, after the vesications break, the ulcerated surface is not disposed to heal, but spreads beyond its original boundary, and becomes extremely painful. As the vesications arise one after another in different places, and are all succeeded by ulcerations, the disease continues with little remission, for several days, generally till the patient expires under the complicated distress arising from pain, loss of sleep, and violent fever.—The children thus affected, are often weak and emaciated, with a dry shrivelled skin. In the few cases which I have seen, the mode of treatment above recommended in the erysipelas of new-born infants, was adopted, but with little success."

Some attempt is made at reconciling the various descriptions of authors, or showing their mistakes in a name that has hitherto been applied to so many vesicular eruptions, the milder kind of which Dr. W. conceives, we think with much justice, were no more than what is often called swine-pox. On the more accurate forms, he offers the following judicious queries:

"1. Whether the case quoted from Seliger is not a case of the erysipelas phlegmonodes, with some incidental variations?"

"2. Whether

"2. Whether in the cases quoted from Sauvages and Thierry, and from the Transactions of the R. Irish Academy, the eruption was not symptomatic, and connected with the typhus or pestilential fever, in the manner stated?"

"3. Whether the disease described by Dr. Langhans was not rather endemic than epidemic or contagious, and referable to some local cause?"

"Pomphylox, (the next division) we are told, is an eruption of bullæ, without any inflammation round them, and without fever. This complaint is termed by Sauvages pemphigus sine pyrexiâ and by Plenk, pemphigus apyretos; but as it differs in the most material points from erysipelas, and from the pemphigus described by nosologists, I have applied to it a new generic title.—The terms pomphos and pompholyx, originally signified an air-bubble, but were afterwards employed in medical language, to express reddish and rounded elevations of the cuticle, containing a watery fluid. Pemphix, from whence is derived the word pemphigus, denoted a small vesicle or phlyctæna."

The author considers three varieties under the terms, p. benignus, p. diutinus, p. solitarius. The first is so trifling, and is ascribed to so many uncertain causes, as scarcely to deserve notice, excepting for the sake of order.

P. diutinus is for the most part connected with other diseases, principally those from fatigue, intemperance, or meagre diet; but in all cases the business of the practitioner seems to be directed principally to the causes and concomitant symptoms.

P. solitarius is attended with sickness, but no fever. The cases described were all in females, one in the left arm, and two in the breast. The excoriations were painful, and attended with hardness, they were readily relieved by Peruvian bark and linseed poultices.

With this ends the first volume of this valuable work. If we have been less unqualified in our praises of the present number, we are willing to impute it more to the intricacy of the subject, than any deficiency in the author; but we are obliged to confess, that useful as the present number certainly is, we have been less satisfied with it than with any of the former.

The number contains title pages and table of contents.

